# Therapeutic Hypothermia Attenuates Cortical Interneuron Loss after Cerebral Ischemia in Near-Term Fetal Sheep

**DOI:** 10.3390/ijms24043706

**Published:** 2023-02-12

**Authors:** Panzao Yang, Joanne O. Davidson, Kelly Q. Zhou, Rani Wilson, Guido Wassink, Jaya D. Prasad, Laura Bennet, Alistair J. Gunn, Justin M. Dean

**Affiliations:** Department of Physiology, Faculty of Medical and Health Sciences, University of Auckland, Auckland 1023, New Zealand

**Keywords:** therapeutic hypothermia, cooling, cerebral cortex, interneuron, EEG, neonatal

## Abstract

Therapeutic hypothermia significantly improves outcomes after neonatal hypoxic-ischemic (HI) encephalopathy but is only partially protective. There is evidence that cortical inhibitory interneuron circuits are particularly vulnerable to HI and that loss of interneurons may be an important contributor to long-term neurological dysfunction in these infants. In the present study, we examined the hypothesis that the duration of hypothermia has differential effects on interneuron survival after HI. Near-term fetal sheep received sham ischemia or cerebral ischemia for 30 min, followed by cerebral hypothermia from 3 h after ischemia end and continued up to 48 h, 72 h, or 120 h recovery. Sheep were euthanized after 7 days for histology. Hypothermia up to 48 h recovery resulted in moderate neuroprotection of glutamate decarboxylase (GAD)^+^ and parvalbumin^+^ interneurons but did not improve survival of calbindin^+^ cells. Hypothermia up to 72 h recovery was associated with significantly increased survival of all three interneuron phenotypes compared with sham controls. By contrast, while hypothermia up to 120 h recovery did not further improve (or impair) GAD^+^ or parvalbumin^+^ neuronal survival compared with hypothermia up to 72 h, it was associated with decreased survival of calbindin^+^ interneurons. Finally, protection of parvalbumin^+^ and GAD^+^ interneurons, but not calbindin^+^ interneurons, with hypothermia was associated with improved recovery of electroencephalographic (EEG) power and frequency by day 7 after HI. The present study demonstrates differential effects of increasing the duration of hypothermia on interneuron survival after HI in near-term fetal sheep. These findings may contribute to the apparent preclinical and clinical lack of benefit of very prolonged hypothermia.

## 1. Introduction

Perinatal hypoxia-ischemia (HI) is a major cause of brain injury in term infants [1,2,3,4]. A common pattern of perinatal HI brain injury involves widespread neuronal injury in the cerebral cortex, including parasagittal watershed zone injury [5,6], which is associated with cognitive dysfunction and learning deficits [7,8]. Gamma-aminobutyric acid (GABA)ergic interneurons are the major inhibitory neurons in the brain and play crucial roles in cortical neuronal network formation during perinatal brain development [9,10,11,12]. Experimental and human studies have shown that cortical interneurons are critical for normal cognitive function, including learning and memory [11,13,14]. Limited human studies have also shown evidence of interneuron disruption associated with neonatal brain injury, including altered interneuron migration, survival, and signaling [13,15,16,17]. Furthermore, we and others have shown that interneurons are particularly sensitive to injury following experimental perinatal HI [18,19,20,21,22,23], with evidence for associated loss of inhibitory control and development of seizures. Thus, disruption of cortical GABAergic interneurons is likely to be a significant contributor to the disability observed after HI in term infants.

Currently the only approved treatment for term infants with moderate to severe hypoxic-ischemic encephalopathy (HIE) is therapeutic hypothermia. If started within 6 h and continued for 72 h, therapeutic hypothermia significantly improves survival and reduces disability [1,24,25,26,27]. However, many infants with moderate to severe HIE still have adverse outcomes despite hypothermia [24,28], raising the possibility that particular cell types may be less protected by current cooling protocols.

Successive animal and clinical studies have investigated whether therapeutic hypothermia protocols can be further optimized [26,29]. In term-equivalent fetal sheep receiving HI induced by 30 min of bilateral carotid artery occlusion, we previously compared longer and shorter durations of hypothermia than current clinical protocols, and found that cooling for 72 h was significantly more beneficial than cooling for 48 h, while extending cooling to 120 h did not further improve overall neuronal survival or electroencephalographic (EEG) recovery [30,31]. It is unknown whether different durations of therapeutic hypothermia may have differential effects on the survival of cortical interneurons after perinatal HI.

In the present study, we tested the hypothesis that 72 h of therapeutic hypothermia would be more effective than 48 h or 120 h of hypothermia for improving the survival of cortical interneurons and neurophysiological function after perinatal HI in term-equivalent fetal sheep.

## 2. Results

### 2.1. Survival of GABAergic Interneurons in the Parasagittal Cortex after Cerebral Ischemia and Therapeutic Hypothermia

**Figure 1:**
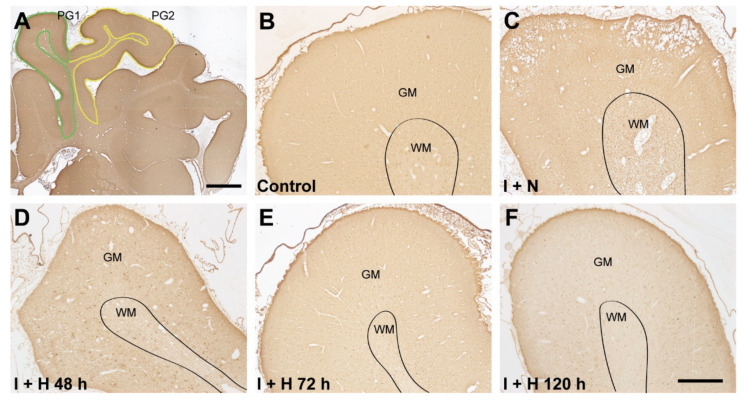
Photomicrographs of glutamate decarboxylase (GAD^+^) interneurons showing the cortices of the first ((**A**); green outline [PG1]) and second ((**A**); yellow outline [PG2]) parasagittal gyri, and the changes of tissue integrity in the PG1 in sham control (**B**), cerebral ischemia + normothermia (I + N; (**C**)), cerebral ischemia + hypothermia 48 h (I + H 48 h; (**D**)), cerebral ischemia + hypothermia 72 h (I + H 72 h; (**E**)), and cerebral ischemia + hypothermia 120 h (I + H 120 h; (**F**)) groups at 7 days recovery. Scale bar: 500 µm.

**Figure 2:**
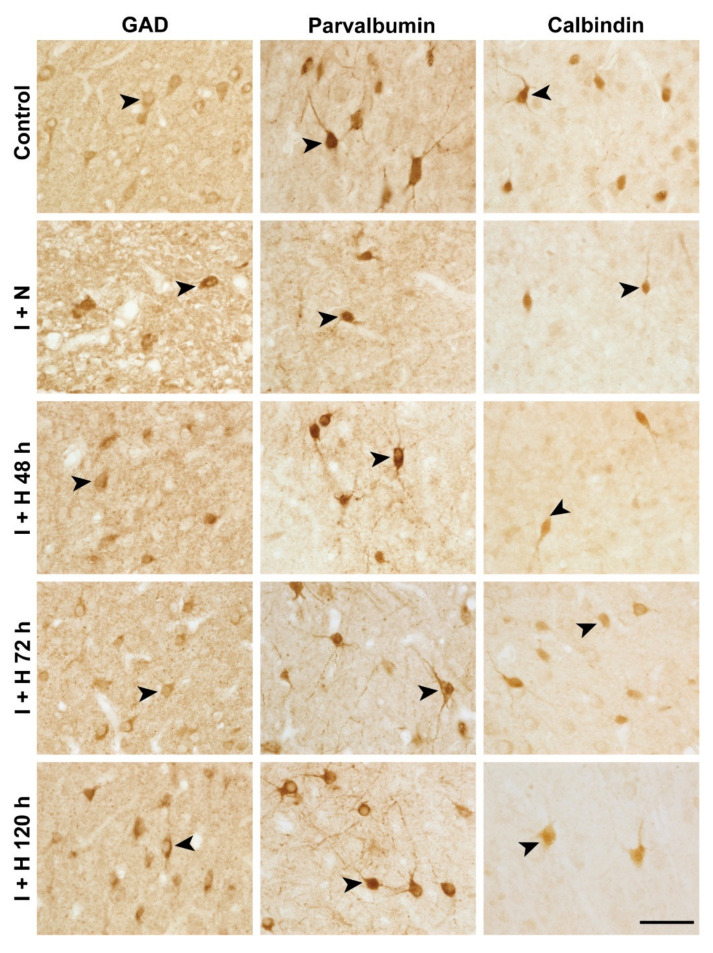
Photomicrographs of glutamate decarboxylase (GAD)^+^, parvalbumin^+^, and calbindin^+^ cortical interneurons in sham control, cerebral ischemia + normothermia (I + N), cerebral ischemia + hypothermia 48 h (I + H 48 h), cerebral ischemia + hypothermia 72 h (I + H 72 h), and cerebral ischemia + hypothermia 120 h (I + H 120 h) groups at 7 days recovery. Black arrowheads show examples of interneurons included in cell counting. Scale bar: 50 µm.

**Figure 3:**
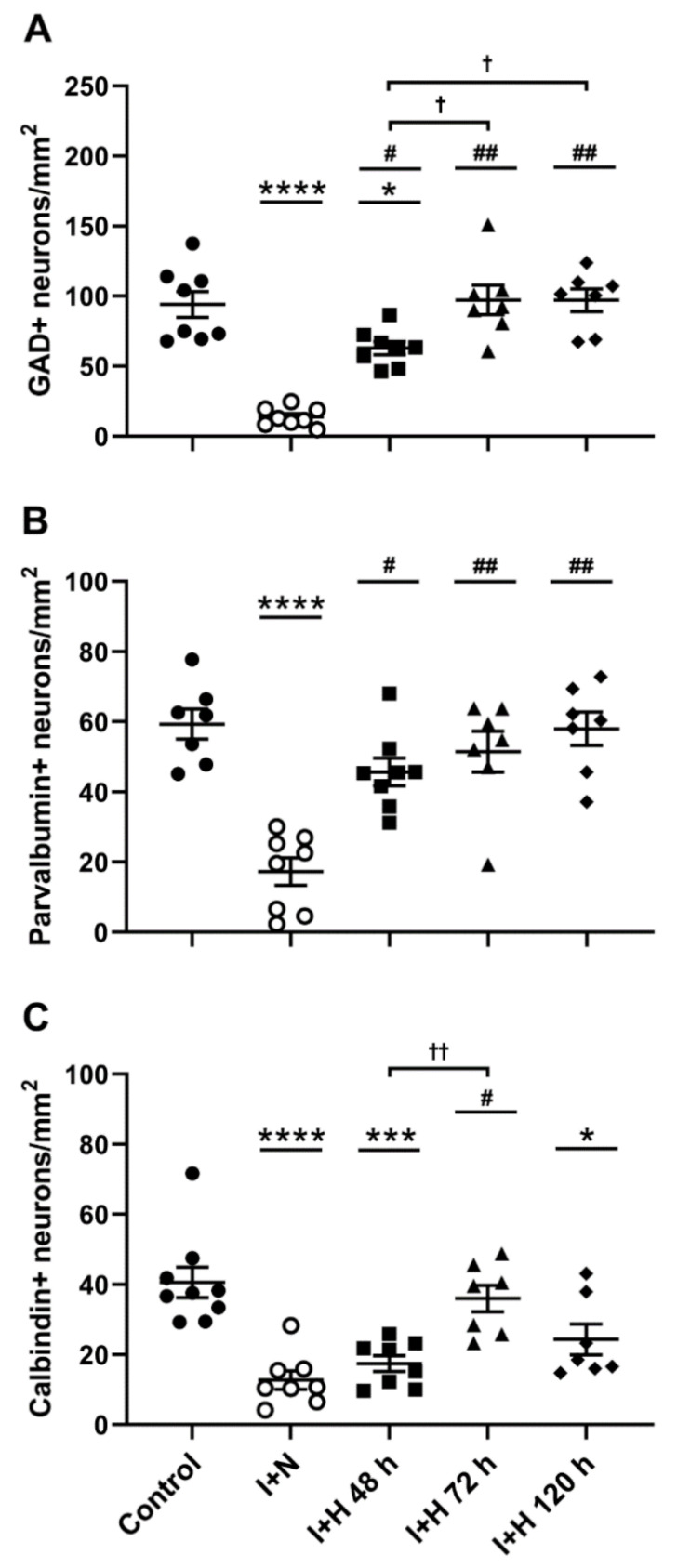
Interneuron survival in the parasagittal cortex in sham control (control), ischemia + normothermia (I + N), ischemia + hypothermia 48 h (I + H 48 h), ischemia + hypothermia 72 h (I + H 72 h), and ischemia + hypothermia 120 h (I + H 120 h) groups at 7 days recovery. Data show densities (cells/mm^2^) of (**A**) glutamate decarboxylase (GAD)^+^, (**B**) parvalbumin^+^, and (**C**) calbindin^+^ interneurons and are presented as mean ± standard error of the mean. Sham control group (closed circles; GAD^+^, n = 8; parvalbumin^+^, n = 7; and calbindin^+^, n = 9). I + N group (open circles; GAD^+^, n = 8; parvalbumin^+^, n = 8; and calbindin^+^, n = 8). I + H 48 h group (closed squares; GAD, n = 8; parvalbumin, n = 8; and calbindin, n = 8). I + H 72 h group (closed triangles; GAD^+^, n = 7; parvalbumin^+^, n = 7; and calbindin^+^, n = 7). I + H 120 h group (closed diamonds; GAD^+^, n = 7; parvalbumin^+^, n = 7; and calbindin^+^, n = 7). * *p* < 0.05, *** *p* < 0.001, and **** *p* < 0.0001 ischemia + normothermia vs. control; # *p* < 0.001 and ## *p* < 0.0001 ischemia + hypothermia vs. ischemia + normothermia; ^†^ *p* < 0.05 and ^††^ *p* < 0.01 comparisons between ischemia + hypothermia groups.

To examine the effect of the length of brain cooling on cortical interneuron survival, we assessed the density of various interneuron populations in the parasagittal cortex (in the first [PG1] and second [PG2] parasagittal gyri; see tracing boundaries in Figure 1A) at 7 d recovery after HI in near-term fetal sheep. Representative low-magnification photomicrographs of the parasagittal cortex stained with glutamate decarboxylase (GAD) in the control, ischemia, and hypothermia groups are shown in Figure 1. Note the general improvement in tissue integrity with 72 h (Figure 1E) and 120 h (Figure 1F) of therapeutic hypothermia compared with the loss of tissue integrity evident in the ischemia + normothermia animals. Representative high magnification images of GAD^+^, parvalbumin^+^, and calbindin-28k^+^ interneurons in the experimental groups are shown in Figure 2 (arrowheads). Cerebral ischemia and therapeutic hypothermia were associated with significant overall changes in interneuron density (Figure 3; GAD^+^, *p* < 0.0001; parvalbumin^+^, *p* < 0.0001; calbindin^+^, *p* < 0.0001; one-way analysis of variance [ANOVA]). Post hoc analysis showed a significant reduction in the density of GAD^+^ (*p* < 0.0001; Figure 3A), parvalbumin^+^ (*p* < 0.0001; Figure 3B), and calbindin^+^ (*p* < 0.0001; Figure 3C) interneurons in ischemia + normothermia animals compared with cell densities in the sham control group.

By contrast, the ischemia + hypothermia 48 h group showed significantly higher densities of GAD^+^ (*p* = 0.0003; Figure 3A) and parvalbumin^+^ (*p* = 0.0006; Figure 3B) interneurons, but not calbindin^+^ (*p* = 0.8831; Figure 3C) interneurons, compared with cell densities in the ischemia + normothermia group. Note that the densities of GAD^+^ and calbindin^+^ interneurons in the ischemia + hypothermia 48 h group remained significantly lower than those in sham controls. Furthermore, the ischemia + hypothermia 72 h group showed complete recovery of all interneuron populations to sham control levels (Figure 3A–C). Finally, the ischemia + hypothermia 120 h group also showed complete recovery of GAD^+^ (Figure 3A) and parvalbumin^+^ (Figure 3B) interneurons, but a significant loss of calbindin^+^ interneurons (Figure 3C).

### 2.2. Relationship between Interneuron Survival and EEG or Spectral Edge Frequency

**Figure 4:**
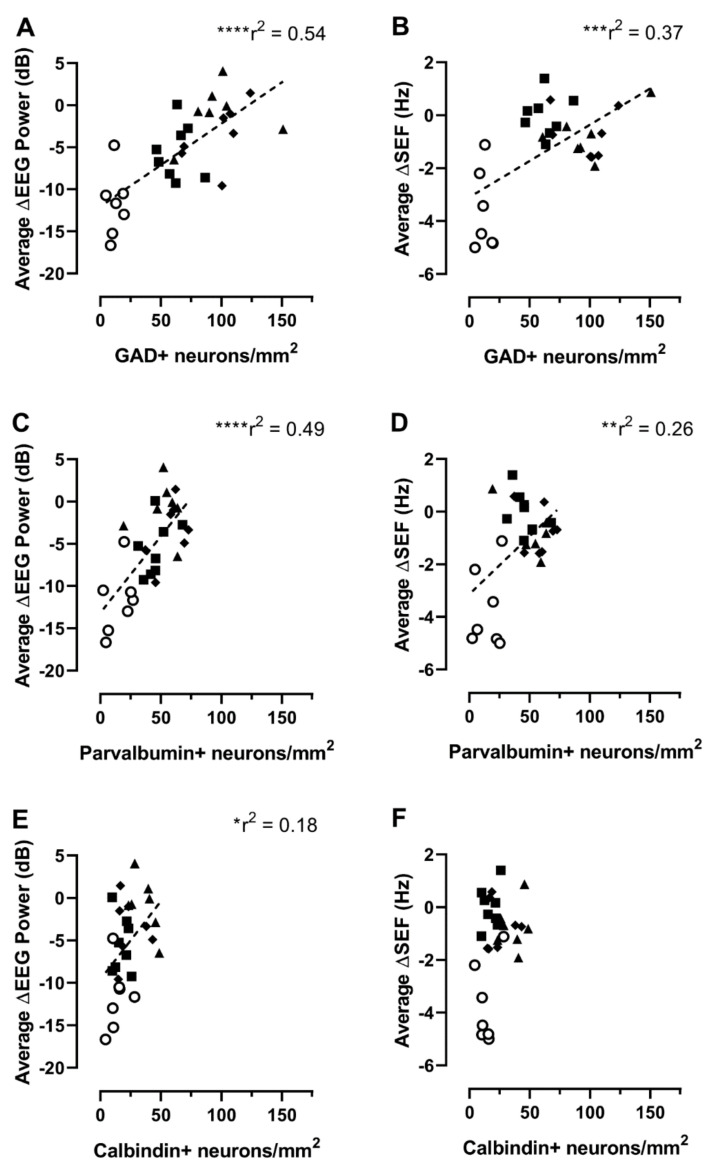
Relationship between cortical interneuron densities and mean electroencephalographic (EEG) power (**A**,**C**,**E**) or spectral edge frequency (SEF) (**B**,**D**,**F**) measured over the last 24 h of recovery. Note that both EEG and SEF were normalized to the baseline period and are presented as ∆EEG and ∆SEF. Ischemia + normothermia group (GAD^+^, n = 7; parvalbumin^+^, n = 7; and calbindin^+^, n = 7; open circles). Ischemia + hypothermia 48 h group (glutamate decarboxylase [GAD]^+^, n = 8; parvalbumin^+^, n = 8; and calbindin^+^, n = 8; closed squares). Ischemia + hypothermia 72 h group (GAD^+^, n = 7; parvalbumin^+^, n = 7; and calbindin^+^, n = 7; closed triangles). Ischemia + hypothermia 120 h group (GAD^+^, n = 7; parvalbumin^+^, n = 7; and calbindin^+^, n = 7; closed diamonds). * *p* < 0.05, ** *p* < 0.01, *** *p* < 0.001, and **** *p* < 0.0001.

Next, we analyzed the relationships between the densities of GAD^+^, parvalbumin^+^, and calbindin^+^ interneurons in the parasagittal cortex with the mean EEG power or spectral edge frequency (SEF) during the last 24 h of recovery in the ischemia + normothermia group and the three ischemia + hypothermia groups. The mean EEG power and SEF showed positive relationships with the densities of GAD^+^ (EEG: r = 0.735, *p* < 0.0001; Figure 4A; SEF: r = 0.610, *p* = 0.0004; Figure 4B), parvalbumin^+^ (EEG: r = 0.700, *p* < 0.0001; Figure 4C; SEF: r = 0.507, *p* = 0.0050; Figure 4D), and calbindin^+^ (note for EEG only: r = 0.426, *p* = 0.0213; Figure 4E; SEF: r = 0.334, *p* = 0.0765; Figure 4F) interneurons.

## 3. Discussion

Cortical interneurons are critical for the normal development and function of the cerebral cortex. There is also increasing evidence that disruption of inhibitory interneuron circuits in the cortex can lead to long-term neurological dysfunction [32,33,34]. Herein, we show differential effects of increasing durations of hypothermia on cortical interneuron survival after HI in near-term fetal sheep. Specifically, hypothermia for 48 h was moderately neuroprotective for both GAD^+^ and parvalbumin^+^ interneurons but did not improve survival of calbindin^+^ interneurons. Consistent with its effects on overall neuronal survival [30,31], hypothermia for 72 h improved all three interneuron phenotypes to sham control levels. By contrast, while hypothermia for 120 h also completely restored numbers of GAD^+^ and parvalbumin^+^ interneurons, it was associated reduced survival of calbindin^+^ neurons. Finally, interneuron protection with hypothermia was associated with functional recovery of cortical EEG by day 7 after HI. Overall, these data suggest that current clinical cooling protocols are also optimal for interneuron survival and that reduced survival of some interneuron populations may contribute to the apparent preclinical and clinical lack of benefit of very prolonged hypothermia.

The marked loss of GAD^+^, parvalbumin^+^, and calbindin^+^ populations of cortical interneurons after near-term HI in the present study is supported by previous reports of reduced survival, growth, and function of cortical interneurons in various animals models of HI brain injury, including in fetal sheep [19,23,35], neonatal rodents [18,20,21,36,37], and preterm baboons [38]. Despite no direct human evidence for cortical interneuron loss after perinatal HI, reduced numbers and complexity of cortical interneurons [15,16], and reduced cortical interneuron migration [17], neurogenesis [39], and GABAergic signaling [13], have been reported in preterm infants.

Therapeutic hypothermia is the only approved treatment for term infants with moderate to severe HIE and can improve survival and reduce disability when started within 6 h and continued for 72 h after injury [1,24,25,26,27]. Nevertheless, 30–50% of these infants treated with therapeutic hypothermia still die or experience significant disability [24,28,40]. A key finding of the present study was that all interneuron populations were protected with this standard clinical cooling protocol, suggesting that any persisting functional deficits in cooled infants may be unrelated to cortical interneuron survival. Furthermore, this interneuron protection was associated with recovery of cortical EEG activity in accordance with the functional role of interneurons in cortical circuits [9,10,11,12,41] and indicative of improved functional outcomes with hypothermia. It should be noted that the correlations between interneuron survival and EEG power were modest and were only observed for the GAD^+^ and parvalbumin^+^ populations. This likely reflects our previous findings of more widespread neuronal and glial cell death in this experimental model, including within multiple populations of neurons in the cerebral cortex, striatum, and hippocampus [42,43,44,45,46], as well as oligodendrocyte cell death and axonal injury in the white matter [46].

We also found reduced interneuron protection with shorter (i.e., 48 h) or longer (i.e., 120 h) durations of cooling, particularly for calbindin^+^ interneurons. A reduced efficacy of 48 h cooling on overall cortical neuronal survival was previously reported following cerebral ischemia in term-equivalent fetal sheep [31]. Additionally, in the same animal model, 120 h cooling provided no added benefit relative to 72 h cooling for overall brain cell survival (e.g., of NeuN-positive neurons and white matter oligodendrocytes) or EEG recovery, with evidence of reduced neuronal survival in some brain regions, including the cerebral cortex [30,31,47]. Supporting this, clinical trials have found no additional benefit of extending cooling to 120 h in term neonates with moderate or severe HIE [29,48].

The reasons for the limited protection of calbindin^+^ interneurons with 48 h cooling relative to GAD^+^ and parvalbumin^+^ interneurons in the present study are unclear. Speculatively, the calbindin^+^ subpopulation may be more sensitive to injury and/or have a different time window of injury evolution, thus reducing the efficacy of cooling therapy. Furthermore, the loss of protection of calbindin^+^ interneurons with 120 h cooling may reflect increased sensitivity to a prolonged, hypothermia-induced suppression of prosurvival or regenerative signaling pathways [49,50,51,52], including molecules released by various populations of microglia or astrocytes in response to injury [52,53,54,55,56,57,58].

GABAergic interneurons account for 10–20% of the total neuron population in the cerebral cortex [33,59] and show a wide morphological, neurochemical, and functional heterogeneity [12,41]. All GABAergic interneurons express GAD, which is required for GABA synthesis. However, in many mammalian species, including humans, there is evidence that the calcium-binding proteins parvalbumin and calbindin are largely distributed within separate interneuron populations in most brain regions, including the cerebral cortex [41,60,61,62,63,64]. Nevertheless, there are contrasting studies showing more cellular overlap of parvalbumin and calbindin labelling in some brain regions in rodents and in humans [65,66,67], including transient regional overlap during development [68]. The differential responses of parvalbumin- and calbindin-labelled cortical interneurons to injury/cooling in the present study support the presumption that these markers identify largely independent populations of interneurons during cortical development.

Parvalbumin-expressing interneurons comprise the majority (~40%) of cortical GABAergic interneurons [69,70] and are distributed across cortical layers 2–6 [12,41,71]. A similar cortical distribution of parvalbumin^+^ interneurons was observed in the present study in fetal sheep. Parvalbumin^+^ interneurons have high spiking rates and dense dendritic fields and innervate pyramidal cells to allow strict temporal and amplitude control to sensory stimuli [72,73,74]. Parvalbumin^+^ interneurons also play important roles in learning and memory by contributing to the establishment of gamma oscillations [75,76,77]. The functions of calbindin^+^ interneurons are less well understood, although recent evidence suggests roles in anxiety, fear-memory, and social behavior in rodents [78]. Note that two populations of calbindin-expressing neurons have been reported in the cerebral cortex: densely stained non-pyramidal neurons and lightly stained pyramidal-like cells [79]. In the present study, we excluded the pyramidal-like cell population (i.e., we only counted calbindin^+^ interneurons) and found that calbindin^+^ interneurons were expressed across all cortical layers in near-term fetal sheep, albeit with a greater localization in the middle layers. Comparatively, a relatively equal distribution of calbindin^+^ interneurons between the upper and lower cortical layers was reported in postnatal day 5 mice [16]. By contrast, calbindin^+^ interneurons were reported to be largely distributed in the infragranular layers of the human visual cortex at term, with a shift to more supragranular distribution with postnatal development [80]. A higher density of calbindin^+^ interneurons in the upper layers was also reported in the prefrontal cortex of the adult monkey [81]. Furthermore, in adult rats, calbindin^+^ interneurons were distributed in layers 2, 3, and 5 of the motor cortex [82]. These contrasting findings may relate to regional and species differences and to changes with development [12,83]. Future studies are required to assess for any cortical layer-specific effects of HI and hypothermia on the various interneuron populations.

## 4. Materials and Methods

### 4.1. Animals and Surgery

All animal procedures in this study were approved by the Animal Ethics Committee of the University of Auckland according to the New Zealand Animal Welfare Act 1999 and the Code of Ethical Conduct for the use of animals for teaching and research, established by the Ministry of Primary Industries, Government of New Zealand. This manuscript complies with the ARRIVE (Animal Research: Reporting of In Vivo Experiments) guidelines for reporting animal research [84].

Time-mated Romney/Suffolk fetal sheep were instrumented by aseptic surgery at 125 days gestation (term is 145 days). Food supply, but not water, was withdrawn 18 h before surgery. Long-acting oxytetracycline (20 mg/kg; Phoenix Pharm, Auckland, New Zealand) was administered to the ewes by intramuscular injection 30 min before surgery. Anesthesia was induced by intravenous administration of propofol (5 mg/kg; AstraZeneca Ltd., Auckland, New Zealand), and, after intubation, general anesthesia was maintained using 2–3% isoflurane in oxygen (Bomac Animal Health, Glendale, NSW, Australia). The depth of anesthesia, maternal heart rate, and respiration were constantly monitored during surgery, and ewes received a constant intravenous infusion of isotonic saline (250 mL/h) to maintain fluid balance.

After a maternal midline abdominal incision, the fetus was exposed, and polyvinyl catheters were inserted into both fetal brachial arteries and the amniotic cavity to measure mean arterial blood pressure. Electrocardiogram electrodes (AS633-3SSF; Cooner Wire, Chatsworth, CA, USA) were sewn across the chest of the fetus for assessment of fetal heart rate. EEG electrodes (AS633-7SSF; Cooner Wire) were fixed on the dura over the parasagittal parietal cortex (10 mm and 20 mm anterior to bregma, and 10 mm lateral) using cyanoacrylate glue, and a reference electrode was sewn over the occiput. A thermistor was fixed over the parasagittal dura (30 mm anterior to bregma) to monitor extradural temperature, and a second thermistor was placed in the esophagus to monitor core body temperature. Inflatable carotid artery occluders were placed around both carotid arteries, after ligating the vertebral–occipital anastomoses. A cooling cap with silicon tubing (3 × 6 mm; Degania Silicone, Degania Bet, Israel) was secured to the head of the fetus. Antibiotics (80 mg gentamicin; Pharmacia and Upjohn, Rydalmere, NSW, Australia) were administered into the amniotic sac before closure of the uterus. The maternal laparotomy skin incision repair was followed by infiltration of 10 mL of 0.5% bupivacaine plus adrenaline (AstraZeneca Ltd., Cambridge, UK) for local analgesia. The maternal long saphenous vein was catheterized to enable postoperative maternal care and euthanasia.

### 4.2. Postoperative Care

Sheep were kept in separate metabolic cages with ad libitum access to food and water in a temperature-controlled room (16 ± 1 °C) with humidity of 50 ± 10% and a 12 h light/dark cycle. Daily antibiotics (600 mg benzylpenicillin sodium and 80 mg gentamicin; Novartis Ltd., Auckland, New Zealand) were administered intravenously to the ewes for 4 days. The fetal catheters were kept unobstructed by continuous infusion of heparinized saline (20 U/mL at 0.15 mL/h), and the maternal catheter was maintained by daily flushing.

### 4.3. Experimental Protocols

Fetuses were randomized to sham control (n = 9), ischemia + normothermia (n = 8), ischemia + hypothermia 48 h (n = 8), ischemia + hypothermia 72 h (n = 7), and ischemia + hypothermia 120 h (n = 8) groups. Cerebral ischemia was induced at 128 ± 1 days of gestation by inflating the bilateral carotid occluder cuffs for 30 min using sterile saline. Successful carotid artery occlusion was indicated by a suppressed EEG activity within 30 s of inflation. Occlusion was not performed on sham control animals. Sham cooling or cooling was initiated at 3 h after the end of occlusion, and continued up to 48 h, 72 h, or 120 h recovery in the ischemia + hypothermia 48 h, ischemia + hypothermia 72 h, and ischemia + hypothermia 120 h groups, respectively. Cooling was performed by circulating cold water through the cooling coil over the fetal scalp using a pump in a cooled water bath to maintain an extradural temperature of 31–33 °C, as previously reported [23,35,46]. In the sham control and ischemia + normothermia groups, no water was circulated so that the cooling coil remained in equilibrium with fetal temperature. After euthanasia, the fetal brains were removed, weighed, and processed for histological studies. Note that the metabolic, electrophysiological, and total neuronal and oligodendrocyte survival data from this cohort of animals was previously reported [30,31,47].

### 4.4. Electrophysiological Data Recording and Analysis

EEG and SEF data were recorded from 24 h before the start of carotid artery occlusion until the end of each experiment. Data were recorded continuously to disk for off-line analysis using custom data acquisition programs (LabView for Windows; National Instruments, Austin, TX, USA). Total EEG power was log transformed (dB, 10 × log [power]) to give a better approximation of the normal distribution. SEF, a measure of the relative frequency of the EEG, was calculated from the power spectrum as the frequency below which 90% of total EEG power resides. Both EEG and SEF were normalized to the baseline period (i.e., ∆EEG and ∆SEF) for analysis. For correlation analyses, the average of the normalized EEG power and SEF was calculated for the last 24 h of the experiment (days 6–7) for each animal.

### 4.5. Tissue Preparation

The fetal sheep brain tissues were prepared as previously reported [23,35,46]. In brief, post-mortem fetal sheep brains were fixed in 10% phosphate-buffered formalin for 1 week at 4 °C, before they were embedded in paraffin. Coronal sections (10-µm thick) were cut using a microtome (Leica Jung RM2035; Leica Microsystems, Auckland, New Zealand). One section taken from the level of the mid-striatum [85] from each animal was used for each analysis (details provided below).

### 4.6. Immunohistochemical Staining

Immunohistochemical staining was performed as previously described [23,35]. All primary and secondary antibodies were diluted in 3% normal goat serum in 0.1 M phosphate buffered saline (PBS). Tissue sections were deparaffinized in 2 × 15 min washes in xylene and then rehydrated in a series of ethanol solutions (100%, 90%, and 75%) for 5 min each, followed by 3 × 5 min washes in PBS. Antigen retrieval was performed in 10 mM citrate buffer (pH 6.0) using the Antigen 200 Retriever (Electron Microscopy Sciences, Emgrid, SA, Australia). Sections were cooled to room temperature and washed for 3 × 5 min in PBS. Endogenous peroxidase activity was inhibited by incubating the sections in 1% hydrogen peroxide in methanol for 30 min, followed by 3 × 5 min washes in PBS. Sections were then blocked in 5% normal goat serum/PBS for 1 h at room temperature before being incubated in primary antibodies (Table 1) overnight at 4 °C. After 3 × 5 min washes in PBS, sections were incubated with biotinylated goat anti-mouse or goat anti-rabbit secondary antibodies (Table 1) for 3 h at room temperature. Sections were then washed for 3 × 5 min in PBS and incubated in ExtrAvidin-Peroxidase (Sigma-Aldrich Co., Saint Louis, MO, USA) diluted in PBS for 2 h at room temperature. After 3 × 5 min washes in PBS, sections were incubated in 3,3′-diaminobenzidine tetrahydrochloride hydrate (Sigma-Aldrich Co.) solution to produce a brown reaction product. The reaction was stopped by washing the tissue sections in distilled water. Sections were then dehydrated in a series of ethanol solutions (75%, 90%, and 100%) for 5 min each, followed by 2 × 10 min washes in xylene. Slides with prepared sections were then cover-slipped with DPX mounting medium (Sigma-Aldrich Co.). Primary control experiments were also performed and showed no positive staining.

### 4.7. Image Analysis

The details for image analysis were previously reported [23,35]. All quantification was performed with the assessor blinded to the treatment groups. In brief, the first (PG1) and second (PG2) parasagittal gyri of the right hemisphere of each brain section (see [23,35] and Figure 1) were traced at 2.5× objective using imaging software (Stereo Investigator; Version 2022.3.1; MBF Bioscience, Williston, VT, USA) connected to a Zeiss AxioImager M2 microscope (Carl Zeiss Microscopy, LLC, Thornwood, NY, USA) equipped with a motorized stage (MAC 6000; MBF Bioscience) [35]. Note that the parasagittal cortex is a watershed region and can develop severe neuronal loss after cerebral hypoperfusion at term [5,6,44], while the fetal EEG in the present study was recorded from the parietal parasagittal cortex. The numbers of DAB-labelled GAD^+^, parvalbumin^+^, and calbindin^+^ interneurons in all layers of the parasagittal cortex in both gyri were counted by transmitted light microscopy at 40× objective using the fractionator probe (Stereo Investigator; grid size = 500 × 500 µm, counting frame size = 150 × 150 µm). For each animal, approximately 60 sites per gyrus were counted. Immuno-positive cells were selected based on the typical patterns of cytoplasm staining and cellular morphologies of different interneuron subtypes (cf. control interneurons in Figure 2) [23,86]. Cells with condensed cytoplasmic aggregates of GAD^+^, parvalbumin^+^, or calbindin^+^ staining were considered injured and were excluded from analysis [23]. The neuronal cell densities (cell number/mm^2^) in the parasagittal cortex (i.e., combined PG1 and PG2) were calculated by dividing the total neuronal numbers with the total area of the counted sites in both gyri. Note that, for some animals, tissue sections for the staining of some interneuron markers were unavailable because of local tissue loss or artifacts (see figure legends for specific animal numbers).

### 4.8. Data Analysis

Differences in neuronal cell densities between the sham control, ischemia-normothermia, ischemia-hypothermia 48 h, ischemia-hypothermia 72 h, and ischemia-hypothermia 120 h groups were analyzed by one-way ANOVA. When there was a significant main effect of treatment (ANOVA, *p* < 0.05), post hoc analysis was performed using Tukey’s multiple comparisons test. Correlations between the numbers of various interneuron populations and ∆EEG or ∆SEF in the ischemia and ischemia + hypothermia groups were analyzed using Pearson correlation coefficients; note that EEG data for the control group were unavailable. A *p*-value < 0.05 was considered statistically significant. All statistical analyses were performed using statistical software (GraphPad Prism v9.2.0; La Jolla, CA, USA). All data are presented as mean ± standard error of the mean.

## 5. Conclusions

In conclusion, the present findings suggest that current clinical therapeutic hypothermia protocols (i.e., started within 6 h and continued for 72 h after injury) may be optimal for reducing cortical interneuron cell death and associated neurophysiological deficits after HIE at near-term. Furthermore, the apparent preclinical and clinical lack of benefit of hypothermia prolonged to 120 h may relate to reduced survival of some interneuron populations.

## Figures and Tables

**Table 1:** Antibodies and markers used for immunohistochemistry.

Antibody	Dilution	Target	Source
**Primary Antibodies**			
Gamma-aminobutyric acid 65/67	1:200	GABAergic interneurons	Abcam, Melbourne, VIC, Australia
Parvalbumin	1:200	Parvalbumin interneurons	Swant Ltd., Marly, Switzerland
Calbindin	1:200	Calbindin interneurons	Swant Ltd.
**Secondary Antibodies**			
Biotinylated goat anti-mouse IgG	1:200		Vector Laboratories, Burlingame, CA, USA
Biotinylated goat anti-rabbit IgG	1:200		Vector Laboratories

## Data Availability

The data presented in this study are available on reasonable request from the corresponding author.
